# Biosynthesis of Phenolic Compounds and Antioxidant Activity in Fresh-Cut Fruits and Vegetables

**DOI:** 10.3389/fmicb.2022.906069

**Published:** 2022-05-25

**Authors:** Wenzhong Hu, Sarengaowa, Yuge Guan, Ke Feng

**Affiliations:** ^1^School of Pharmacy and Food Science, Zhuhai College of Science and Technology, Zhuhai, China; ^2^School of Food and Health, Zhejiang Agricultural and Forestry University, Hangzhou, China; ^3^LiveRNA Therapeutics Inc., Zhuhai, China; ^4^College of Life Science and Technology, Huazhong Agricultural University, Wuhan, China

**Keywords:** fresh-cut fruits, fresh-cut vegetables, biosynthesis, phenolic compounds, antioxidant activity

## Abstract

Phenolic compounds are secondary metabolites and widely distributed in higher plants. When plants are subjected to injury stress, the rapid synthesis of more phenols is induced to result in injury defense response for wound healing and repair. Fresh-cut fruits and vegetables undergo substantial mechanical injury caused by pre-preparations such as peeling, coring, cutting and slicing. These processing operations lead to activate the biosynthesis of phenolic compounds as secondary metabolite. Phenolic compounds are important sources of antioxidant activity in fresh-cut fruits and vegetables. The wound-induced biosynthesis and accumulation of phenolic compounds in fresh-cut fruits and vegetables have been widely reported in recent years. This article provides a brief overview of research published over the last decade on the phenolic compounds and antioxidant activity in fresh-cut fruits and vegetables. It is suggested that fresh-cut processing as mechanical wounding stress can be used as an effective way to improve the nutritional composition and function of fresh-cut produces.

## Introduction

Phenolic compounds are widely found in various amounts in fruits, vegetables, cereals, and beverages such as wine, coffee, cocoa, and tea (Papuc et al., 2017). Phenolic compounds are secondary metabolites from plant. It has the function with reducing reactive oxygen species and inhibiting lipid peroxidation in human (Fenga, 2016; Villarreal-García et al., 2016; Hu et al., 2021). It also has high pharmaceutical value and other biological activities as the main contributors to the total antioxidant capacity of fruits and vegetables. The main phenolic substances include monophenol, phenolic acid, hydroxyl cinnamic acid derivatives and flavonoids in plant. Flavonoids include also flavonols, anthocyanins, and isoflavones. Phenols has some biological and pharmacological activities function including the antioxidant, anti-inflammatory, anti-tumor, anti-viral and anti-allergic substances with potential health benefits, especially in the prevention and treatment of chronic diseases in humans, including neurodegenerative and diabetes diseases, prostate cancer and cardiovascular diseases (Bahadoran et al., 2013; Sahpazidou et al., 2014; Nikolaos et al., 2015). The survey shows the higher daily intake with proportion of fruit and vegetables can induce lower risk of disease including mental disorders, cardiovascular disease and various cancers in the aspect of health and nutrition survey (Stanner et al., 2004; Lee et al., 2019). Some studies have also shown that fresh fruits and vegetables contain rich phenolic compounds. There were important health benefits for maintaining human health, increasing the body’s resistance to oxidative damage, preventing disease including cardiovascular diseases and cancer by moderate intake of phenolic compounds in daily diets (Chu et al., 2002; Reyes et al., 2007; Wolfe and Liu, 2008; Costa et al., 2017). The chemical compounds with function of pharmaceutical and nutraceutical have been studied in plant in these years (Becerra-Moreno et al., 2012). Plants as the biofactory of phenolic compounds could produce much more phenolics by simply increasing wounded stress treatment and intensity (Liu, 2004; Becerra-Moreno et al., 2012). Therefore, wounding stress is an effective method which increase phenolic content of postharvest fruits and vegetables. Fresh-cut processing operation induces significantly increase in phenolic content and antioxidant capacity of these fresh-cut fruits and vegetables, such as fresh-cut carrot, fresh-cut potato, fresh-cut onion and fresh-cut dragon fruit, indicating that it was universal phenomenon of synthetic accumulation of phenols in fresh-cut fruits and vegetables induced by mechanical wounding stress (Surjadinata and Cisneros-Zevallos, 2012; Berno et al., 2014; Torres-Contreras et al., 2014; Li et al., 2017).

## Phenolic Compounds in Fresh-Cut Fruits and Vegetables

Fresh-cut fruits and vegetables were also known as semi-processed fruits and vegetables, minimally processed fruits, and vegetables. Fresh-cut fruits and vegetables have the advantage of fresh, convenience, ready-to-eat, and ready-to-use. There are more numerous phenolic compounds which are considered as the main contributors to the total antioxidant capacity of fruits and vegetables (Li et al., 2019; Guan et al., 2021a). The cutting activates the biosynthesis of phenolic compounds of fresh-cut fruits and vegetables, which defend and heal the wounding damage at the injured site or site adjacent (Reyes and Cisneros-Zevallos, 2003; Cisneros-Zevallos and Jacobo-Velazquez, 2020). Many studies reported significant increases in content of phenolic compounds and antioxidant activity after wounding in different types of fresh-cut fruits and vegetables such as lettuce, celery, mushroom, broccoli, carrot onions, and mangoes (Vina and Chaves, 2006; Oms-Oliu et al., 2010; Surjadinata and Cisneros-Zevallos, 2012; Zhan et al., 2012; Benito Martinez-Hernandez et al., 2013; Maribel Robles-Sanchez et al., 2013; Berno et al., 2014; Torres-Contreras et al., 2014). The cutting processing of fresh-cut produces induces the rapid synthesis and accumulation of phenolics in short time. The accumulation of phenolic compounds may improve the functional value of these fresh-cut products. Table 1 showed the differences in phenolic contents of whole and fresh-cut fruits and vegetables.

**TABLE 1:** Comparison of phenolic contents of whole and fresh-cut fruits and vegetables.

Fruits and vegetables types	Content of phenolics (mg 100 g^–1^)	Fresh-cut fruits and vegetables	Content of phenolics (mg 100 g^–1^)	Increase times	References
Carrot	48	Fresh-cut carrot	250	5.21	Surjadinata and Cisneros-Zevallos, 2012
Potato	50	Fresh-cut potato	95	1.90	Becerra-Moreno et al., 2012
Dragon	602	Fresh-cut dragon	1,250	2.08	Guan et al., 2021a
Broccoli	150	Fresh-cut broccoli	280	1.87	Martínez-Hernández et al., 2011
Onion	298	Fresh-cut onion	451	1.51	Berno et al., 2014
Lettuce	20	Fresh-cut lettuce	29	1.45	Reyes et al., 2007
Celery	23	Fresh-cut celery	26	1.13	Reyes et al., 2007
Sweet potato	90	Fresh-cut sweet potato	110	1.22	Reyes et al., 2007

According to the above results, fresh-cut carrots, fresh-cut dragon fruits, fresh-cut broccoli have 5.2 times, 2.1 times, and 1.9 times than the content of whole fruit (Surjadinata and Cisneros-Zevallos, 2012; Torres-Contreras et al., 2014; Guan et al., 2021a). Therefore, wound-induced can be used as an effective way to improve the nutritional composition of fresh-cut produces (Jacobo-Velázquez et al., 2011). The greater the wound area of fresh-cut fruits and vegetables, the greater the damage intensities to cells of fruit and vegetable, which will lead to a series of more significant changes in physiological, and biochemical. These physiological changes and reactions will cause rapid increase in metabolic reactions such as rapid increase in respiration, decline in quality, loss of fresh quality, especially in the accumulation of phenolics. The cutting styles affect the accumulation of phenolic compounds which have bioactive function in broccoli. It indicated that broccoli was considered as resource of promoting more health nutrient properties when broccoli was treated with certain cutting intensities (Torres-Contreras et al., 2017). The phenolic content of dragon fruit with different cutting types increased significantly within 2 days, i.e., the whole fruit increased by 34%, the slice increased by 63%, the half slice increased by 78% and the equal slice increased by 90% (Li et al., 2017). Table 2 showed the differences in contents of phenolic compounds in fresh-cut fruits and vegetables with different cut-wounding intensities.

**TABLE 2:** Comparison contents of phenolic compounds in fresh-cut fruits and vegetables with different cut-wounding intensities.

Fruits and vegetables	Cutting styles	Content of phenolic (mg 100 g^–1^)	Increase times	References
Broccoli	Florets 10 × 10 cm	54.95	1.27	Torres-Contreras et al., 2017
	Florets 5 × 5 cm	56.77	1.31	
	Florets 2.5 × 2.5 cm	63.29	1.46	
	Shreds	65.59	1.51	
Carrot	Slices	39.04	1.67	Guan et al., 2020a
	Pies	60.94	2.60	
	Shreds	112.9	5.21	
Onion	Slices	47.23	1.35	Jacobo-Velázquez and Cisneros-Zevallos, 2009
	Pies	50.06	1.44	
	Shreds	57.87	1.66	
Pitaya	Slices	1012.31	1.43	Li et al., 2017
	Half-slice	1130.19	1.59	
	Quarter-slice	1250.35	1.76	

According to Table 2, the content of phenols is 54.59 mg 100 g^–1^ in fresh-cut roccoli florets (10 × 10 cm) tissues, up to 65.59 mg 100 g^–1^ in the shreds tissue. The content of phenolic compounds is 39.04 mg 100 g^–1^ in the fresh-cut carrot slices, up to 57.87 mg 100 g^–1^ in the shreds tissue. It indicated that the content of phenolic substances in fresh-cut fruits and vegetables was enhanced with cutting intensities produced by different cutting styles.

## Biosynthesis of Phenolic Compounds in Fresh-Cut Fruits and Vegetables

Phenolic compounds in wounded fresh-cut fruits and vegetables are produced quickly in part to produce lignin as wounding stress response. The activation of phenylpropanoid metabolism and the content of phenolic compounds can be stimulated with different cutting intensity (Jacobo-Velázquez et al., 2015). Some factors including wounding intensity, cut methods, storage temperature, and gas composition can promote the mechanism during storage time (Padda and Picha, 2008; Jacobo-Velázquez and Cisneros-Zevallos, 2009; Surjadinata and Cisneros-Zevallos, 2012; Pérez-López et al., 2018).

It has been proven that the primary metabolites of plant as defense compounds, carbon source and signaling molecules can induce the increasing with secondary metabolite products (Jacobo-Velázquez et al., 2015). The study demonstrated that the increasing of some bioactive compounds involving with phenolic compounds and glucosinolates of broccoli was enhanced with the method of cutting (Torres-Contreras et al., 2017).

It was reported the rapid production of reactive oxygen species (ROS) after fresh cutting of dragon fruit, as well as the subsequent improvement of phenylalanine ammonia-lyase (PAL) activity and the accumulation of phenolics, proved that ROS as a signaling molecule induced and initiated the operation of phenylpropane metabolic pathways and the synthesis of phenolics in fresh-cut fruits and vegetables (Jacobo-Velázquez et al., 2011; Li et al., 2017; Torres-Contreras et al., 2017; Guan et al., 2020a). ROS as signaling molecules play an important role in increasing of phenolic compounds of cutting carrot tissues (Padda and Picha, 2008; Torres-Contreras et al., 2014). ROS are produced with the occurrence of wounded plant tissue and its action is regulating wounded defense response. Some reports have demonstrated that primary metabolism lays the foundation for secondary metabolism in fruits and vegetables stimulated by wounding stress. The content of chlorogenic acid isomers in damaged potato tubers is accompanied by a significant increase in reducing sugar levels, indicating a close relationship between primary metabolism of carbohydrate and secondary metabolism of phenylalanine (Torres-Contreras et al., 2014). The increase of shikimic acid and phenylalanine which are related to the primary metabolism, cause the producing of individual phenolic compounds in carrot tissues treated with cutting (Liu, 2004; Becerra-Moreno et al., 2015). Furthermore, the generation of signal was activated by fresh-cutting operation, which induced PAL activity to synthesis more phenolic compounds. The activity of PAL, a key enzyme of phenylpropane metabolic pathway, affects the accumulation of phenolic compound generated by phenylalanine among the phenylpropane metabolic pathway. The promotion of PAL enzyme in wounded tissues would to enhance cellular antioxidant activity of fresh-cut product (Bozin et al., 2008). As shown in Table 3, the enhancement of PAL activity and total phenols content was greater as the intensity of wounding increased with different cutting styles (Table 3). Cutting or minimally processed can enhance nutrition of fresh fruits and vegetables, such as garlic and ginger must be mechanical mashed or chopped to induce the production of more phenolics and bactericidal action, and can undergo some reactions of biochemical in plant *in vivo* to produce special flavor substances (Nuutila et al., 2003; Benkeblia, 2004; Cardelle-Cobas et al., 2005; Queiroz et al., 2009; Guan et al., 2020b). Rapid response induced by fresh cutting of fruits and vegetables not only induces physiological and biochemical changes such as large number of wounded ethylene production and rising respiratory rate, but also by activating defense gene transcription and expression in plant cells, synthetic defense enzyme system, including superoxide dismutase (SOD), catalase (CAT), ascorbate peroxidase (APX), lipoxgenase (LOX), polyphenol oxidase (PPO), peroxidase (POD). These enzymes can scavenge reactive oxygen free radical to prevent its attack on the cell membrane and then avoid membrane lipid peroxidation. Wounding stress also induces the activation defense response genes in fresh-cut fruits and vegetables, and synthesizes and accumulates more phenolic substances in their injured sites and their adjacent sites, such as chlorogenic acid, ferulic acid, caffeic acid, lignin, and other secondary metabolites (Saltveit, 2004; Reyes et al., 2007). These secondary metabolites are biosynthesized by using essential carbon sources and frameworks mainly from the glycolytic pathways of phosphoenolpyruvate and pentose phosphate in the process of oxygen uptake decomposition of sugar. The plant produces a series of signaling substances in its body after being subjected to mechanical stress-wounded signaling molecules, which activate the transcription and expression of functional genes in the local and undamaged sites or away from the injured sites, mainly including the generation, transfer, induction, signal transduction and amplification of the injured signaling molecules, and ultimately the expression of the wound-induced genes (Li et al., 2018).

**TABLE 3:** Comparison of PAL enzyme activity of fresh-cut fruits and vegetables with different cutting styles.

Fruits and vegetables	Cutting styles	PAL enzyme activity (U kg^–1^)	Increase times	References
Broccoli	Florets 10 × 10 cm	54.95	1.27	Guan et al., 2020a
	Florets 5 × 5 cm	56.77	1.31	
	Florets 2.5 × 2.5 cm	63.29	1.46	
	Shreds	65.59	1.51	
Carrot	Slices	39.04	1.67	Surjadinata and Cisneros-Zevallos, 2012
	Pies	60.94	2.60	
	Shreds	112.9	5.21	
Onion	Slices	560	3.73	Han et al., 2016
	Pies	790	5.27	
	Shreds	1,060	7.06	
Pitaya	Slice	150.32	1.43	Li et al., 2017
	Half-slice	167.13	1.70	
	Quarter-slice	184.99	1.86	

In general, mechanical cutting processing induces wounded tissue of fresh-cut fruit and vegetable to accelerate the respiratory oxidation of sugar-primary metabolism, such as glucose metabolism, respiratory metabolism, and energy metabolism in short time. But the relationship between the primary metabolism and wound-induced secondary metabolism and the precursor of phenolic compound synthesis and how much energy is required to provide are rarely reported. There are few studies on the mechanism and regulation of phenolics induced by mechanical wounded stress after cutting, especially the relationship between primary metabolism and secondary metabolism, molecular biology-transcriptional, proteomics, and metabolomics analyses of phenylpropane metabolic pathways induced by mechanical wounded stimulation signal (Hodges and Toivonen, 2008; Han et al., 2017a; Li et al., 2018).

**FIGURE 1:**
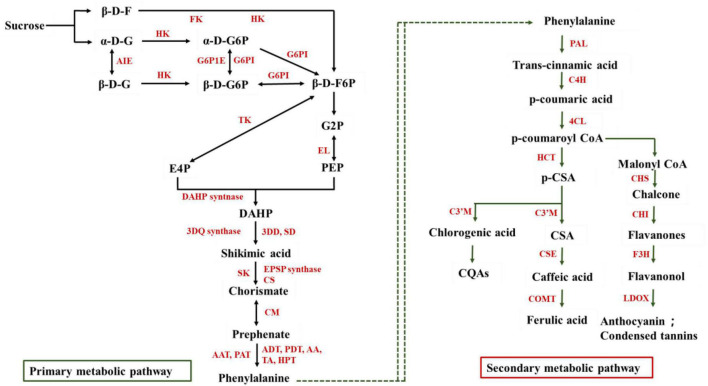
The biosynthesis pathways of phenolics in fresh-cut fruits and vegetables. β-D-F, β-D-fructose; α-D-G, α-D-glucose; β-D-G, β-D- glucose; α-D-G6P, α-D-glucose 6-phosphate; β-D-G6P, β-D-glucose 6-phosphate; β-D-F6P, β-D-fructose 6-phosphate; G2P, 2-phosphoglycerate; PEP, Phosphoenolpyruvate; E4P, Erythrose 4-phosphate; DAHP, 3-deoxy-D-arabino-heptulosonic Acid 7-Phosphate; CQAs, Caffeoylquinic acid; p-CSA, p-coumarin shikimic acid; CSA, Coumarin shikimic acid. FK, Fructose kinase; HK, Hexose kinase; G6P1E, Putative glucose-6-phosphate 1-epimerase; AIE, Aldose 1-epimerase; G6PI, glucose-6-phosphate isomerase; TK, transketolase; EL, enolase; DAHP syntnase, Phospho-2-dehydro-3-deoxyheptonate aldolase syntnase; 3DQ synthase, 3-dehydroquinate synthase; 3DD, SD, 3-dehydroquinate dehydratase shikimate dehydrogenase; SK, Shikimate kinase; EPSP synthase, 5-enolpyruvylshikimate-3-phosphate synthase; CS, Chorismate synthase; CM, Chorismate mutase; AAT, PAT, Arogenate dehydratase/prephenate dehydratase; ADT, PDT, Bifunctional aspartate aminotransferase and glutamate/aspartate-prephenate aminotransferase; AA, Aspartate aminotransferase; TA, Aminotransferase; HPT, Histidinol-phosphate aminotransferase; PAL, Phenylalanine ammonia-lyase; C4H, Cinnamate 4-hydroxylase; 4CL, 4-coumarin coenzyme A ligase; HCT, Hydroxycinnamoyl transferase; C3’M, Cytochrome; CSE, Caffeoylshikimate esterase; COMT, Bergaptol O-methyltransferase; CHI, Chalcone isomerase; F3H, Flavanone-3-hydroxylase; LDOX, Leucoanthocyanidin dioxygenase.

The biosynthesis pathways of phenolics in fresh-cut fruits and vegetables were showed in Figure 1. In plants, glycolysis and pentose phosphate pathways can provide carbon source for the synthesis of secondary metabolites, including phenolic compounds (Hodges and Toivonen, 2008). During the process of cutting, the tissue of fruits and vegetables will produce kinds of injury signals and then further accelerate the oxidative decomposition of sugar into erythrose 4-phosphate (E4P) and phosphoenolpyruvate (PEP) (Dixon and Paiva, 1995; Jacobo-Velázquez and Cisneros-Zevallos, 2012). With the catalytic action of enzymes, E4P and PEP could degrade to produce shikimic acid and phenylalanine, which ended the primary metabolic process. The phenylalanine accumulation from the primary metabolic process provides precursors for the synthesis of phenolic compounds through phenylpropane metabolism. For example, the wounding stress up-regulated the genes including the conversion of reducing sugar to phenylalanine phenolic compound in fresh-cut carrots (Han et al., 2017b). Phenylalanine could convert into phenols, phenolic acids, anthocyanins, flavonoids, isoflavones, and other polyphenolic metabolites with several enzymatic reactions. Phenylpropane metabolism includes several branches such as the central metabolic pathway, anthocyanin, flavonoid, and isoflavone synthesis pathways. In the central metabolic pathway, 4-coumarin coenzyme A ligase (4CL), cinnamate 4-hydroxylase (C4H), and phenylalanine ammonia-lyase (PAL) were considered the critical enzymes, which could catalyze phenylalanine into phenolic acids such as shikimic acid, caffeic acid, chlorogenic acid, and ferulic acid (Li et al., 2017). It was reported that PAL, C4H, and 4CL enzymes activities were increased obviously which contributed to the result of catechin, hydroxybenzoic acid, chlorogenic acid, caffeic acid, sinapic acid, and cinnamic acid accumulation in fresh-cut broccoli (Guan et al., 2019). Furthermore, similar results were founded in fresh-cut onion, lettuce, carrot, celery, and pitaya (Vina and Chaves, 2006; Reyes et al., 2007; Surjadinata and Cisneros-Zevallos, 2012; Berno et al., 2014; Li et al., 2017). p-coumaroyl CoA could be used as an important precursor to enter the flavonoid metabolism and further product flavanones, flavanonol, anthocyanin, and other phenols (Torres-Contreras et al., 2018). Therefore, more antioxidant activities, drug-active substances, health promoters could be activated though cutting or wounding stress. That is a convenient way to keeping healthy and physiology in business and at home. Meanwhile, according to different biological characteristics and cutting styles, could make suggestions on cutting method of fresh-cut fruits and vegetables suitable for application of commercial and domestic.

## Antioxidant Activity of Phenolic Compounds in Fresh-Cut Fruits and Vegetables

The accumulation of secondary metabolites in plant with wound-induced can be applied as effective method to produce more bioactive compounds in fresh-cut fruits and vegetables, which have significant health function and antioxidant effects because they are rich in phenolics, anthocyanins, carotenoids, vitamins, and other antioxidant substances. For instance, fresh-cut carrots respond to wounding stress producing and accumulating caffeoylquinic acids, which are phenolic compounds (PC) with potential applications in the treatment and prevention of chronic-diseases including hepatitis B, diabetes, obesity, cardiovascular diseases, neurodegenerative diseases, and HIV (Surjadinata and Cisneros-Zevallos, 2012). It has been reported that the antioxidant activity of vitamin C in apple is less than 0.4% of the antioxidant activity of whole fruit (Cheong et al., 2002). The phenolics in fruits and vegetables have much more antioxidant capacity than that of vitamin C (Santana-Gálvez et al., 2019). When the balance between oxidants and antioxidants in the human body is destroyed by many free radicals, it can lead to oxidative stress and DNA damage, making human cells cancerous (Costa et al., 2017). The antioxidants such as phenols in fruits and vegetables mainly maintain human health and prevent cancer by reducing oxidative damage, scavenging free radicals, inhibiting oxidative stress response, and reducing the probability of cardiovascular disease, coronary heart disease, and stroke (Aune et al., 2017; Guan et al., 2021b). Common fruits and vegetables such as apples, spinach, cabbage, red peppers, onions and broccoli have been shown to have significant inhibitory effects on hepatocellular carcinoma cells in HepG2, also suggesting that about 32% of cancers can be prevented by diet change (Gillman et al., 1995; Eberhardt et al., 2000; Chu et al., 2002).

Cutting damage can induce broccoli, carrots, potatoes, onions, dragon fruit, celery, lettuce, to produce a large number of phenolic compounds, resulting in increase of antioxidant activity of fresh-cut fruits and vegetables (Saltveit, 2004; Vina and Chaves, 2006; Martinez-Hernandez et al., 2013; Berno et al., 2014; Torres-Contreras et al., 2014; Han et al., 2017b; Li et al., 2017). The prevention of chronic diseases such as obesity, diabetes, hepatitis, cardiovascular disease, neurodegenerative diseases, and HIV have potential applications by using fresh-cut products (Robinson et al., 1996; Morton et al., 2000; Balasubashini et al., 2004; Kim et al., 2005; Thom, 2007; Wang et al., 2009). It has been proved that the cellular antioxidant activity of flower cluster, flower, 1/2 flower, and shred broccoli was reached at 1.15, 1.20, 1.24, and 1.41 μmol QE kg^–1^, which was increased by 52.7, 59.2, 64.8, and 86.5% compared to the whole broccoli, respectively, which indicated that the nutrients of fruits and vegetables could be enhanced through fresh cutting and preprocessing (Bozin et al., 2008). Meanwhile, the kinds of different individual phenolic compounds have the effect on antioxidant activity, it has been reported that caffeic acid, cinnamic acid, and sinapic acid contribute to the increase of the antioxidant activity of wounded broccoli (Bahadoran et al., 2013), whereas, the quercetin, caffeic acid and vanillic acid were the critical carrier of antioxidant activity in fresh-cut onion (Padda and Picha, 2008).

Moreover, the content of phenols compound and antioxidant capacity in fresh-cut products could be affected obviously with different cutting intensities. Studies on fresh-cut broccoli and pitaya have demonstrated that total phenols content and the ability of scavenging reactive oxygen species *in vitro* was enhanced with cutting intensity increase (Martinez-Hernandez et al., 2013; Li et al., 2017). However, this result was inconsistent with the results of a previous study in fresh-cut red cabbage and potato, which indicated that the increase in antioxidant capacity after wounding depends on the type of fruit or vegetable tissue (Reyes et al., 2007). These studies suggested that the application of fresh-cut processing operations as postharvest wounded stress, whether commercially or in the home, is an easy way to get more healthy, physiological and drug-active phenolic substances and antioxidant activity.

## Author Contributions

WH: conceptualization, writing (original draft preparation), supervision, and project administration. Sarengaowa and KF: investigation. Sarengaowa and YG: resources. Sarengaowa, YG, and KF: writing (review and editing). All authors contributed to manuscript revision, read, and approved the submitted version.

## Conflict of Interest

KF was employed by company LiveRNA Therapeutics Inc. The remaining authors declare that the research was conducted in the absence of any commercial or financial relationships that could be construed as a potential conflict of interest.

## Publisher’s Note

All claims expressed in this article are solely those of the authors and do not necessarily represent those of their affiliated organizations, or those of the publisher, the editors and the reviewers. Any product that may be evaluated in this article, or claim that may be made by its manufacturer, is not guaranteed or endorsed by the publisher.
